# Effectiveness of Caregiver Training in Mindfulness-Based Positive Behavior Support (MBPBS) vs. Training-as-Usual (TAU): A Randomized Controlled Trial

**DOI:** 10.3389/fpsyg.2016.01549

**Published:** 2016-10-06

**Authors:** Nirbhay N. Singh, Giulio E. Lancioni, Bryan T. Karazsia, Jeffrey Chan, Alan S. W. Winton

**Affiliations:** ^1^Department of Psychiatry and Health Behavior, Medical College of Georgia, Augusta UniversityAugusta, GA, USA; ^2^MacTavish Behavioral HealthRaleigh, NC, USA; ^3^Department of Neuroscience and Sense Organs, University of BariBari, Italy; ^4^Department of Psychology, The College of WoosterWooster, OH, USA; ^5^Movement for the Intellectually Disabled of Singapore, SingaporeSingapore; ^6^Department of Psychology, Massey UniversityPalmerston North, New Zealand

**Keywords:** Mindfulness-Based Positive Behavior Support, MBPBS program, 1:1 staffing, aggressive behavior, physical restraints, psychological stress, staff turnover, benefit-cost analysis

## Abstract

Caregivers of individuals with intellectual and developmental disabilities (IDD) often end up having their medical and psychological well-being compromised due to the stressful nature of caregiving, especially when those in their care engage in aggressive behavior. In this study, we provided caregivers with mindfulness-based training to enable them to better manage their psychological well-being and, through this, to also enhance specific indices of quality of life of the individuals in their care. Thus, the aim of the present study was to evaluate in a randomized controlled trial (RCT) the comparative effectiveness of Mindfulness-Based Positive Behavior Support (MBPBS) and Training-as-Usual (TAU) for caregivers in a congregate care facility for individuals with severe and profound IDD. The comparative effects of the two training conditions were assessed in terms of caregiver variables care recipient variable (number of aggressive events), and agency variables Results showed that MBPBS was significantly more effective than TAU in enabling the caregivers to manage their perceived psychological stress, and to reduce the use of physical restraints and stat medications for aggressive behavior of the individuals in their care. In addition, there were significant reductions in aggressive events by the individuals in their care, 1:1 staffing of individuals with aggressive behavior, and staff turnover. Furthermore, the MBPBS training was significantly more cost-effective than the TAU training. If replicated in future RCT studies, MBPBS may provide an effective means of enhancing socially acceptable bidirectional engagement of caregivers and care recipients within a person-centered context.

## Introduction

Caregivers, regardless of whether they are unpaid family members or paid staff, often end up having their psychological well-being compromised due to the stressful nature of caregiving (McIntyre et al., 2002; Hastings et al., 2006; Herring et al., 2006). Many change jobs because of psychological stress (Hastings and Beck, 2004) and burnout (Chung and Harding, 2009), or require therapy if they continue in their role of caregiving. In this context, psychological stress results from emotional and physiological reactions to job-related demands that a caregiver is unable to cope with, and burnout results when prolonged stress exhausts the physical and emotional strength of the caregiver. Recent research suggests that caregivers can enhance the self-management of their psychological stress by engaging in a disciplined practice of meditation (Singh, 2014). There are several mechanisms that may come into play when a caregiver regularly practices meditation, especially mindfulness (Anālayo, 2016). For example, enhanced mindfulness may provide caregivers better emotional self-regulation during periods of acute stress. It may also increase cognitive flexibility, when their responses are informed by awareness of what is unfolding in the present moment without the distortions of their own emotions and perceptions of the events. Every time caregivers are able to view each unfolding event with a beginner’s mind, perceiving each event as if it is occurring for the first time, then “right action” emerges (Suzuki, 1970).

Caregivers of individuals with intellectual and developmental disabilities (IDD) face additional stress from the severe challenging behaviors of individuals in their care (Hensel et al., 2012; Didden et al., 2016). For example, individuals with IDD evince aggression that is often of low frequency but high intensity, and are likely to physically hurt their caregivers as well as their peers. The prevalence rate of aggression in this population varies considerably, ranging from about 7% (Emerson et al., 2001) to over 50% (Tenneij et al., 2009; Jahoda et al., 2013). Current research suggests that when caregivers are stressed due to the aggressive behavior of the individuals in their care, they tend to develop a negative attitude toward the individuals, eventually leading to negatively interacting with them or avoiding them (Jahoda et al., 2013). Indeed, caregiver stress may lead them to recommend that individuals in their care who are aggressive be treated with restrictive procedures such as psychotropic medications, emergency medications, and physical restraints (Singh et al., 2011a; Deveau and McGill, 2014).

In response, agencies have typically taken one of three general approaches to assist caregivers in delivering services to individuals with IDD. In the most widely used approach, caregivers are provided additional training in managing the challenging behaviors of individuals in their care. This could take many forms. For example, caregivers often receive new employee and in-service training in behavior management procedures, typically involving the principles and practice of positive behavior support (PBS; MacDonald, 2016; Morris and Horner, 2016). When implemented with fidelity, PBS has been shown to be immensely successful in caregiver management of the challenging behaviors of individuals with IDD. However, PBS procedures may not be used with fidelity in actual practice because of staff shortages, the inability of staff to use the procedures with more than one person at a time, caregiver stress, and the intensity of effort required (Allen et al., 2005; Didden et al., 2016). While assisting caregivers to better manage the aggressive behavior of the individuals is a viable and logical approach, it does not help the caregivers to manage their own stress and burnout.

A second approach involves providing training that enables caregivers to better manage their work-related psychological distress. For example, Noone and Hastings (2009, 2010) showed that when caregivers participate in Promotion of Acceptance in Carers and Teachers training, it enables them to significantly decrease their psychological distress even when faced with a slight increase in occupational stress. This training included three key components of Acceptance and Commitment Therapy (Hayes et al., 1999)—acceptance, cognitive mindfulness, and values clarification. In a similar approach, Brooker et al. (2013) showed that when caregivers participate in occupational mindfulness (OM) training, they are able to decrease their stress, enhance psychological well-being, and increase job satisfaction. The OM training includes mindfulness practices, aspects of positive psychology (e.g., signature strengths; Seligman, 2002), and various cognitive therapy exercises.

A third approach is to enhance the ability of the caregivers to skillfully use behavior management strategies and to learn ways of reducing their own occupational stress. For example, Singh et al. (2009) added a mindfulness-based training to the existing training in behavior management principles for caregivers, and demonstrated positive changes in the behaviors of both the caregivers and the individuals in their care. Brooker et al. (2014) essentially replicated these results using a different set of mindfulness-based training procedures. In a small multiple-baseline design study, Singh et al. (2015) evaluated the effects of an integrated mindfulness-based training with PBS training (i.e., Mindfulness-Based Positive Behavior Support [MBPBS]; Singh et al., 2016a). The results suggested that the MBPBS training enabled the caregivers to greatly reduce their psychological stress, eliminate staff turnover, and substantially reduce and then eliminate the use of physical restraints with individuals who evinced aggressive behavior. In a proof-of-concept quasi-experimental design study, Singh et al. (2016b) further evaluated the effectiveness of MBPBS training for caregivers. The results corroborated earlier findings of lowered caregiver psychological stress and staff turnover, and significantly less use of physical restraints. Furthermore, in both studies, a benefit-cost analysis showed substantial financial savings for the agency due to their staff participating in the MBPBS training (Singh et al., 2015, 2016b).

These studies strongly suggest that caregivers can learn to regulate their emotions more effectively through MBPBS training than with standard agency in-service training. Furthermore, enhanced caregiver emotional regulation appears to have benefits for the caregivers (e.g., reduced psychological stress and staff turnover), as well as for those in their care (e.g., reduced engagement in aggressive behavior). These studies focused on caregivers who provided services to individuals with IDD who functioned at mild to moderate levels but did not include those who functioned at severe and profound levels. In addition, these studies did not use control groups to compare the effects of the different training using a robust experimental design. Thus, the aim of the present study was to evaluate the comparative effectiveness of MBPBS and Training-as-Usual (TAU) for caregivers in a congregate care facility for individuals with severe and profound IDD, using a randomized controlled trial (RCT). The comparative effects of the two training conditions were assessed in terms of caregiver variables (i.e., use of physical restraints, use of stat [emergency] medications, perceived psychological stress), a care recipient variable (i.e., number of aggressive events), and agency variables (i.e., 1:1 staffing of individuals with aggressive behavior, staff turnover, benefit-cost of the two trainings).

## Materials and Methods

### Setting

The study was conducted at a large congregate care facility for individuals with IDD. The agency served individuals who were at the severe and profound levels of functioning and exhibited varying levels of challenging behaviors (e.g., physical aggression, property destruction, pica, rumination, stereotypy). The individuals resided in six homes, with each home accommodating between 6 and 10 individuals (mean = 8 per home). In total, there were 48 long-term beds, all of which were filled except when an individual needed short-term admission at a local hospital for acute medical care. All individuals exhibited challenging behaviors, but only 34 of the 48 individuals exhibited these behaviors at a level of severity and/or frequency that required the caregivers to implement formal behavior management plans. Of the 34 with formal behavior management plans, up to 10 of them required 1:1 staffing on any given day for aggressive behavior toward staff and/or peers. A 1:1 staffing level was used for the safety of the individual, staff and peers.

### Participants

**FIGURE 1:**
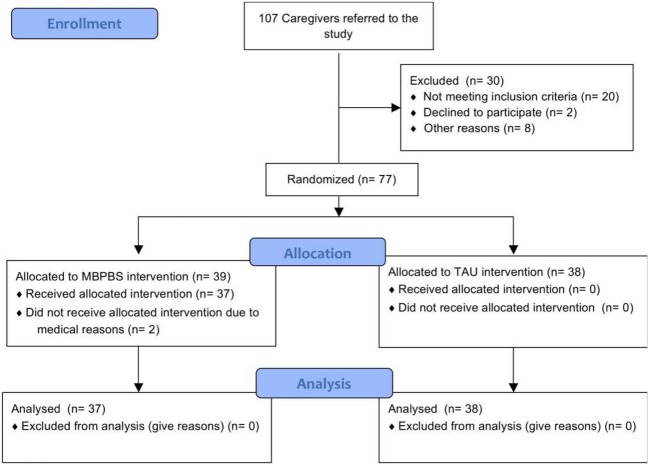
**Participant flow through the trial (CONSORT diagram)**.

As a part of the facility’s consumer engagement plan for enhancing the health and wellness of individuals with IDD, the entire caregiver staff was required to receive additional in-service training. All 107 caregivers were enrolled, of which 30 did not meet the inclusion criteria (i.e., full-time employment, consent to participate in the training, and availability during the training). Using a random number generator, the remaining 77 caregivers were randomized into MBPBS or TAU conditions. Of the 39 caregivers randomized to the MBPBS group, 2 dropped out before the study due to personal reasons (i.e., one due to change of job and the other due to late stage pregnancy). None of the 38 caregivers randomized to the TAU group dropped out. **Figure 1** presents a CONSORT participant flow diagram.

The sociodemographic data for both the caregivers and the individuals with IDD in their care are presented in **Table 1**.

**Table 1:** Socio-demographic characteristics of the caregivers and individuals with IDD in their care for the Mindfulness-Based Positive Behavior Supports (MBPBS) and Training-as-Usual (TAU) conditions.

	MBPBS		TAU	
	Caregivers	Individuals with IDD	Caregivers	Individuals with IDD
Number of participants	37	24	38	24
Mean age in years (SD)	43.05 (10.39)	39.21 (7.61)	45.08 (7.87)	42.33 (9.22)
Age range (years)	23–62	27–54	25–59	24–57
Gender: males	14 (37.83%)	16 (66.67%)	10 (26.32%)	16 (66.67%)
Level of functioning				
Severe	na	9 (37.5%)	na	7 (29.17%)
Profound	na	15 (62.5%)	na	17 (70.83%)
Number of individuals on psychotropic medications	na	20 (83.33%)	na	19 (79.16%)
Number of individuals with mental illness	na	20 (83.33%)	na	19 (79.16%)
Number of individuals with behavior plans for aggressive behavior	na	18 (75%)	na	16 (67%)

*na, not applicable.*

### Ethics Statement

All training procedures in the study were in accord with the 1964 Helsinki declaration and its later amendments or comparable ethical standards. Written informed consent was obtained from all caregivers who participated in the study. The provider agency, caregivers, and facility review committee approved the MBPBS and TAU training and data collection.

### Procedure

#### Experimental Design

A RCT was conducted to evaluate the comparative effectiveness of MBPBS vs. TAU on caregiver, individuals with IDD, and agency variables. Unless otherwise stated, standard agency data collection procedures were followed as part of the study protocol.

#### Interventions

##### Mindfulness-Based Positive Behavior Supports

The standard 7-day MBPBS protocol, as reported by Singh et al. (2015, 2016b), was used. The training was presented in three parts, spread over a 10-week period. Part I lasted one 8-h day, Part II was five 8-h days (i.e., 40 h), and Part III was one 8-h day. All 37 caregivers in this condition received all the MBPBS training in a group format. **Table 2** presents the MBPBS program and a brief outline of each day’s training.

**Table 2:** Outline of the 7-day MBPBS program.

PART I	
**Day 1**	
(First 1-day training)	Samatha meditation
	Kinhin meditation
	Vipassanā meditation
	Five hindrances—sensory desire, ill will, sloth and torpor, restlessness and remorse, and doubt
	Daily logs and journaling

**PART II**	

**Day 2**	Review of meditation practice
(First day of 5-day intensive training)	Introduction to the Four Immeasurables (*Brahmavihara*: *metta*—lovingkindness; *karuna*—compassion; *mudita*—empathetic joy; *upekkha*—equanimity)
	Equanimity meditation
	Beginner’s mind
	Applications to PBS practice

**Day 3**	Review of day 2 instructions and practices
	Further instructions on the Four Immeasurables
	Equanimity meditation
	Lovingkindness meditation
	Being in the present moment
	Applications to PBS practice

**Day 4**	Review of days 2 and 3 instructions and practices
	Further instructions on the Four Immeasurables
	Equanimity meditation
	Lovingkindness meditation
	Compassion meditation
	The three poisons—attachment, anger, and ignorance
	Applications to PBS practice

**Day 5**	Review of days 2 to 4 instructions and practices
	Further instructions on the Four Immeasurables
	Equanimity meditation
	Lovingkindness meditation
	Compassion meditation
	Joy meditation
	Attachment and anger—shenpa and compassionate abiding meditations
	Applications to PBS practice

**Day 6**	Review of days 2 to 5 instructions and practices
	Review and practice Samatha, Kinhin, and Vipassanā meditations
	Review of the Four Immeasurables
	Practice equanimity, lovingkindness, compassion, and joy meditations
	Attachment and anger—meditation on the soles of the feet
	Review of applications to PBS practice
	Review of the MBPBS training program

**PART III**	

**Day 7**	
(Second 1-day training)	Review of the meditation instructions and practices (daily logs)
	Review and practice Samatha, Kinhin, and Vipassanā meditations
	Review of the Four Immeasurables
	Practice equanimity, lovingkindness, compassion, and joy meditations
	Emotion regulation and anger—meditation on the soles of the feet Instructions for practicing three ethical precepts—refrain from (a) harming living creatures, (b) taking that which is not given, and (c) incorrect speech
	Applications to PBS practice
	Review of the 7-day MBPBS training program

Part I was on the first day of the first week of training, during which the caregivers received instructions in and practiced three foundational meditations: Samatha, Kinhin, and Vipassanā. The caregivers received in-depth instructions and practiced the fundamentals of meditation posture. They were instructed to sit comfortably with a straight spine, without slouching or stretching the shoulders, with their head tilted slightly forward, eyes slightly open or closed, with the tip of their tongue lightly touching the upper palate, the right hand resting over the left hand on the lap, with thumbs just touching, and breathing evenly (Buksbazen, 2002). They were taught to focus on their breathing, without deliberately changing the length of each breath. They learned to count an inhalation and exhalation as one breath until they reached 10 breaths, before restarting the counting cycle. They were taught to simply observe their discursive thoughts and emotions, without interacting with them or trying to suppress them. That is, they were required to focus their awareness on whatever took place in their mind without judgment or engagement. Samatha meditation is the foundational meditation that provides the practitioner with the stability of mind on which to build all other meditation practices. In addition, they were taught Kinhin (walking) meditation and Vipassanā (insight) meditation (McDonald, 2005). Kinhin is a walking meditation that enables a person to be in the present moment while walking slowly and mindfully. Vipassanā meditation is used to gain insight into the true nature of reality through mindfulness of breathing, thoughts, feelings, and actions (Shonin et al., 2015). Toward the end of the first day of training, all caregivers were instructed to develop a personal meditation practice, beginning with a few minutes each day and incrementally increasing it until they reached between 20 and 30 min of daily practice. Finally, they were required to log their daily meditation practice. The caregivers were required to practice the three meditations daily until training in Part II that was scheduled for the fifth week.

During Part II (days 2 to 6 of training), the caregivers received instructions on the nature of the Four Immeasurables (equanimity, lovingkindness, compassion, and empathetic joy), and meditation practices in equanimity, lovingkindness, compassion, and empathetic joy (Kyabgon, 2004). They also received instructions on the concepts and application of the beginner’s mind (Suzuki, 1970), being in the present moment, the three poisons—attachment, anger and ignorance (Kyabgon, 2004), shenpa and compassionate abiding (Chödrön, 2007, 2010; Kongtrül, 2008), meditation on the Soles of the Feet (Singh et al., 2011b), and the general concept of emotion regulation and application of the various meditations in the caregivers’ work and private life.

Part III was scheduled on the first day of the 10th week (i.e., seventh full day of training), for follow-up, wrap-up, and follow-through meditation practices. This involved further meditation practice, review of the caregivers meditation practices and experiences, questions and answers from the group, and how the caregivers would continue their practice till the end of week 40—when formal aspects of the study concluded—and beyond.

For the PBS component of the MBPBS training, Part I was devoted to ascertaining the current knowledge of the caregivers in the principles and practice of PBS, and collaboratively developing a training program in PBS within the context of mindfulness-based practices. The PBS training program was informed by current literature on PBS (Morris and Horner, 2016) and staff training in PBS (MacDonald, 2016), the caregivers’ lived experience of working with individuals with IDD who periodically engaged in high-intensity but low frequency aggressive behavior, and the seamless interface with the caregivers’ personal practice of mindfulness. During Part II (i.e., the 5-day training), the caregivers were instructed in the following five components of standard PBS plans: setting event strategies, preventive strategies, teaching strategies, consequence strategies, and quality of life outcomes (Lucyshyn et al., 2015). In terms of interfacing with their mindfulness practices, they were given instructions on mindful observation of the individual’s behavior, mindful communication (with a focus on mindful prompting and feedback), mindful pause between requests and prompts, and mindful use of reinforcement contingencies that focused on the rate, quality, magnitude, delay, and specificity of the reinforcement delivered contingently and non-contingently to the individuals with IDD in their care (Singh et al., 2016a). Part III (i.e., the seventh full day of training), involved a review of the mindfulness-based PBS practice, discussion of the need for formal PBS programs, questions and answers regarding the MBPBS practice, and follow-through PBS practices.

##### Training-as-Usual

The TAU in-service training followed the same three-Part, 7-day training timeline as in the MBPBS training. The training staff of the provider agency provided this training. It covered the following general areas of applied behavior analysis: definitions and characteristics; principles, processes and concepts; behavioral assessment; evaluation of outcomes; development and implementation of behavior management plans; and ethical considerations in using a behavioral approach to interventions (Cooper et al., 2007; Mayer et al., 2012). The caregivers were given instructions in reading, understanding, and implementing behavior management plans, observing the implementation of behavior support plans by expert staff, role playing implementation of PBS plans, and getting feedback on their implementation efforts. Further, they discussed their current behavior support plans, their effectiveness, fidelity of implementation, data collection, graphing the data, and revisions based on the data.

#### Training Adherence

For both training conditions, the caregivers’ attendance at the 7-day training program was documented. In addition, caregivers in both training conditions were requested to record the time they spent in daily meditation during the 40 weeks of the study. All caregivers in both training conditions attended and fully participated in the 7 days of training. The daily logs showed that all 37 caregivers in the MBPBS training condition began their practice of meditation following Part I training (i.e., first 1-day training) and continued throughout the 40 weeks of the study. The duration of meditation gradually increased from a few minutes during Part I and averaged 15 min by the end of training in Part II (i.e., the 5-day training). There was a further increase following the 5-day training, and this averaged 33 min by the end of training in Part III. Thereafter, the caregivers averaged between 25 and 40 min of daily meditation, with occasional meditation holidays. Overall, on average, the caregivers in the MBPBS condition meditated for 89% (range: 0–96%) of the days. Two caregivers from the TAU training condition had a personal meditation practice prior to the study and both continued their meditation practice during the 40 weeks of the study. On average they meditated between 20 and 30 min daily, with occasional breaks from meditation.

#### Trainers

The MBPBS trainer was an experienced behavior analyst at the BCBA-D level, with over 35 years of hands-on experience in developing and implementing behavior support plans. In addition, the trainer had a 40-year personal meditation practice and experience in the mindful delivery of services in behavioral health. Segments of training in Parts I, II, and III were videotaped and 10 randomly selected segments of 10–15 min from each day of the 7-day training (i.e., 70 training segments) were rated for fidelity of training by another qualified meditation trainer who was also an expert in PBS. The fidelity of the MBPBS training was rated at 100% for both the meditation instructions and the training in PBS.

The TAU trainer was an experienced behavior analyst at the BCBA level, with over 20 years of training experience in behavior management. This trainer did not have a personal meditation practice. As with the MBPBS training, segments of training in Parts I, II, and III were videotaped and 10 randomly selected segments of 10–15 min from each day of the 7-day training (i.e., 70 training segments) were rated for fidelity of training by another qualified trainer in behavior management. The fidelity of the behavioral training was also 100%.

#### Measures

##### Aggressive Events

An aggressive event was defined as an individual hitting, biting, scratching, punching, kicking, slapping, or destroying property. Staff recorded each instance of an aggressive event on an incident reporting form at the point of occurrence and this was later entered in the facility’s incident management database. By policy, each incident was double-checked by the home supervisor for occurrence and accuracy of reporting. The reliability of reporting and logging the occurrence of aggressive events was 98% (range: 94–100%).

##### Physical restraints

A physical restraint was defined as a brief physical hold of an aggressive individual by a caregiver when there was imminent danger of physical harm to the individual, peers or staff, and the behavior could not be controlled with verbal redirection. Staff recorded each instance of the use of a physical restraint at the point of occurrence and this was later entered in the facility’s risk management database. By policy, each use of physical restraint was double-checked by the home supervisor for occurrence and accuracy of reporting. The reliability of reporting and logging the occurrence of physical restraints was 100%.

##### Stat medicine

Stat medicine is prescribed during medical, psychiatric or behavioral emergencies for the immediate safety of the individual. Stat medication was defined as an emergency medication prescribed for the immediate calming of an individual who was aggressive and could not be managed by other means, including physical restraints. Each prescription was counted as one event as recorded by a registered nurse in the individual’s Medication Administration Record (MAR). Only those prescriptions that were prescribed specifically as emergency medication for aggressive behavior were counted.

##### One-to-one staffing

A 1:1 staffing is the level of supervision used when an individual needs close attention for a specific reason and it is designed to ensure the safety of the individual, peers or staff. For the purpose of this study, 1:1 staffing was defined as the level of enhanced observation ordered by a physician or psychologist for an individual with IDD who evinces aggressive behavior. Each individual’s treatment team determined the need for level of supervision, the nursing administration assigned the staff, and the home manager ensured the provision of level of supervision on a shift-by-shift basis. Level of supervision staff was recorded as being present for the assigned duties 100% of the time.

##### Staff stress

Caregivers in both training conditions completed the Perceived Stress Scale-10 (PSS-10; Cohen et al., 1983) as a measure of perceived stress at two time points: on the first and last day of the 40-week study. The PSS-10 provides an index of the degree to which people perceive their lives as stressful and indicates how often they have found their lives to be unpredictable, uncontrollable, and overloaded in the last month. This rating scale includes items such as, “In the last month, how often have you found that you could not cope with all the things that you had to do?” The caregivers responded to 10 questions on a five-point scale, ranging from 0 (never) to 4 (very often). Their responses were summed to create a psychological stress score, with higher scores indicating greater psychological stress. The PSS-10 has adequate psychometric characteristics (Cohen and Williamson, 1988). In the present study, Cronbach’s alpha was 0.82, indicating good reliability.

##### Staff turnover

The facility’s Human Resources Department provided the staff turnover data, which included all instances of any staff member leaving the employment of the agency due to staff injury on the worksite during the 40 weeks of the study period. Data were extracted only for the fulltime staff involved in the MBPBS and TAU training conditions.

##### Cost effectiveness data

The facility’s Finance Department provided cost data on (1) work days lost due to staff injury, (2) instances of 1:1 staffing, (3) staff needing medical and physical rehabilitation therapy due to injury, (4) staff resigning due to staff injury who were replaced, (5) staff required for MBPBS and TAU training, and (6) temporary staff required during MBPBS or TAU training. All costs were included, regardless of whether the costs were borne by the agency or by workers’ compensation.

### Data Analyses

The effectiveness of the MBPBS and TAU conditions were examined in several ways. For several variables, the unit of analysis was a count variable for an entire condition, not for individuals within a condition. These examples included the number of aggressive events per week, the number of uses of physical restraints per week, the number of emergency stat medicine prescriptions per week, and the number of additional 1:1 staffing needed per week. As these variables are not at the individual level, they do not lend themselves to traditional analyses for RCTs. Therefore, we employed two strategies to examine change across time within conditions and differences across groups.

First, we were able to examine change across time within each condition by treating each group as an *n* of 1. In doing so, we plotted the count of each variable for each condition across all weeks of the study. Second, we averaged counts per week across the 10-week Training phase and the 30-week post-training phase, respectively, for each condition. The resulting *M*’s (with *SD*’s) could then be compared across groups by phase with independent samples *t*-test (and corresponding Cohen’s *d* values as effect size measures). Using paired samples *t*-test, we compared change across phases within each condition. Note that alternative approaches, such as use of a mixed-model ANOVA, were not possible because the unit of analysis was not individuals.

The data on perceived stress were unique to individuals. Therefore, we used a mixed-model ANOVA to compare main effects of condition, time, and their interaction. Effect sizes reported include η^2^ for an overall effect size, and Cohen’s *d*’s for direct comparisons for a specific phase across conditions, or for a specific condition across time.

## Results

### Demographic Variables

We ran a series of Chi-Square and Independent Samples *t*-test to compare demographic characteristics of participants in the MBPBS and TAU conditions. There were no statistical differences between the groups (all *p*’s > 0.05).

### Caregiver Variables

#### Perceived Stress

**FIGURE 2:**
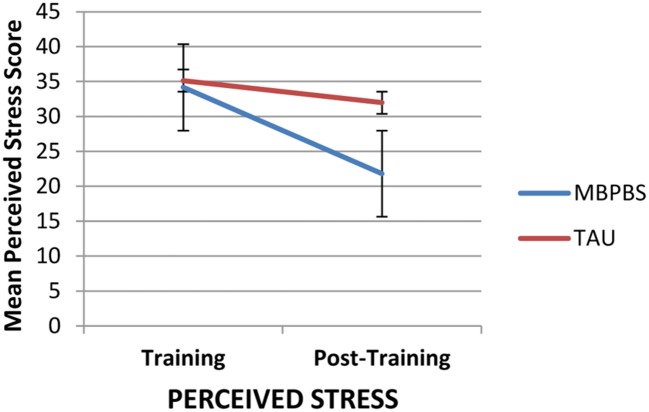
**Ratings of caregiver perceived stress on PSS-10 on the first day of training and the last day of post-training in the two conditions, Mindfulness-Based Positive Behavior Supports (MBPBS) and Training-as-Usual (TAU).** Note that higher scores indicate greater psychological stress. Error bars report standard error of the mean.

**Figure 2** shows there was a decrease in the perceived stress score from the first day of Training (Time 1) to the last day of post-training (Time 2) of 36.15% in the MBPBS condition and 9.02% in the TAU condition. Differences across time and between conditions on PSS were examined with a 2 (condition: MBPBS vs. TAU) × 2 (timepoint: pre vs. post) mixed model ANOVA. The results revealed a significant interaction, *F*(1,73) = 73.70, *p* < 0.001 (η^2^ = 0.50). **Figure 2** shows there was no significant difference between groups on the first day of Training (pre) phase (Cohen’s *d* = 0.36), but there was a significant difference, with a large effect size, on the last day of the post-training phase (Cohen’s *d* = 2.78). The within-groups effect sizes from pre to post for the MBPBS and TAU conditions were as follows: Cohen’s *d* = 2.60 and 0.70, respectively.

#### Physical Restraints

**FIGURE 3:**
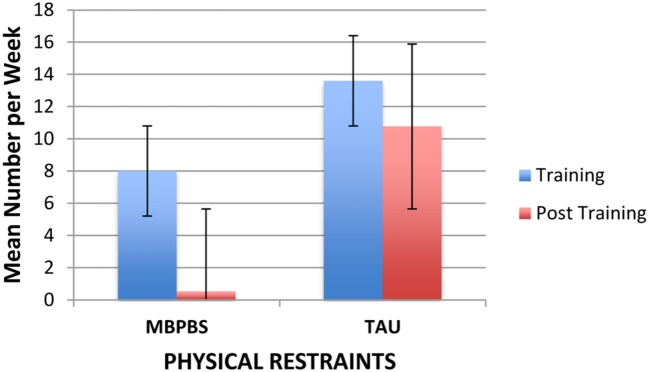
**Mean number of physical restraints per week used by caregivers contingent on aggressive behavior of the individuals in the MBPBS and TAU conditions.** Error bars report standard error of the mean.

As evident in **Figure 3**, the weekly use of physical restraints decreased substantially from the Training to the post-training phase for the MBPBS condition, while there was no such decrease in the TAU condition. On average, the use of physical restraints in the MBPBS condition was 8.00 (range = 2–13) per week during the Training phase and 0.53 (range = 0–3) in the post-training phase. Similarly, on average, the use of physical restraints in the TAU condition was 13.60 (range = 9–17) per week during the Training phase and 10.77 (range = 5–16) in the post-training phase. During the Training phase, there was a statistically significant difference in physical restraint use per week across the MBPBS (*M* = 8.00, *SD* = 4.06) and TAU conditions (*M* = 13.60, *SD* = 2.72), *t*(18) = 3.62, *p* = 0.002 (Cohen’s *d* = 1.62). During the post-treatment phase, use of physical restraints was lower in the MBPBS condition (*M* = 0.53, *SD* = 0.90) than in the TAU condition (*M* = 10.77, *SD* = 3.21), *t*(58) = 1.73, *p* = 0.10 (Cohen’s *d* = 4.34).

#### Stat Medication

**FIGURE 4:**
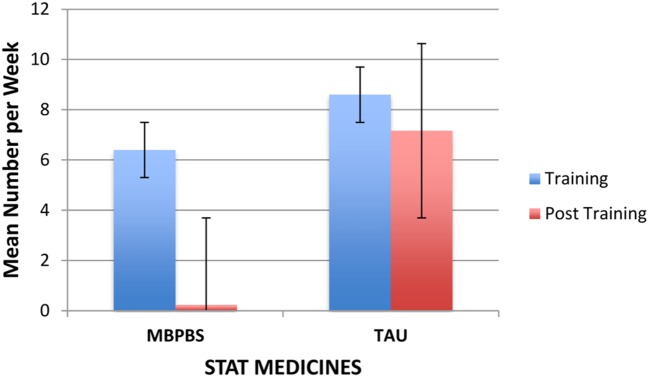
**Mean number of stat medicines per week used by caregivers contingent on aggressive behavior of the individuals in the MBPBS and TAU conditions.** Error bars report standard error of the mean.

As shown in **Figure 4**, the weekly use of stat medications decreased substantially from the Training to post-training phase for the MBPBS condition, while there was no such decrease in the TAU condition. On average, the use of stat medication in the MBPBS condition was 6.40 (range = 2–11) per week during the Training phase and 0.23 (range = 0–3) in the post-training phase. Similarly, on average, the use of stat medication in the TAU condition was 8.60 (range = 5–12) per week during the Training phase and 7.17 (range = 2–14) in the post-training phase. During the Training phase, there was no statistically significant difference in mean use of stat medications per week between the MBPBS (*M* = 6.40, *SD* = 3.24) and TAU conditions (*M* = 8.60, *SD* = 2.37), *t*(18) = 1.73, *p* = 0.10 (Cohen’s *d* = 0.77). During the post-training phase, the use of stat medications in the MBPBS condition was significantly lower (*M* = 0.23, *SD* = 0.68) than in the TAU condition (*M* = 7.17, *SD* = 3.30), *t*(58) = 11.28, *p* < 0.001 (Cohen’s *d* = 2.91).

### Care Recipient Variable

#### Aggressive Events

**FIGURE 5:**
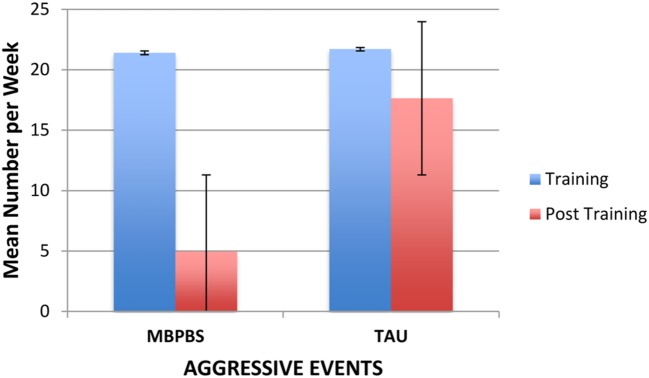
**Mean number of aggressive events per week exhibited by the individuals in the MBPBS and TAU conditions.** Error bars report standard error of the mean.

**Figure 5** shows the number of aggressive events by each training condition. On average, in the MBPBS condition there were 21.75 (range = 19–27) aggressive events per week during the Training phase and 5.91 (range = 0–21) in the post-training phase. Similarly, on average, in the TAU condition there were 22.25 (range = 17–26) aggressive events per week during the Training phase and 17.75 (range = 10–24) in the post-training phase. There was no significant difference between the MBPBS (*M* = 21.40, *SD* = 2.76) and TAU conditions (*M* = 21.70, *SD* = 3.71), *t*(18) = 0.21, *p* = 0.84 (Cohen’s *d* = 0.09) during the Training phase. However, the difference between conditions was significant in the post-training phase, *t*(58) = 11.01, *p* < 0.001 (Cohen’s *d* = 2.84), with the MBPBS condition (*M* = 4.97, *SD* = 4.86) demonstrating fewer aggressive events than the TAU condition (*M* = 17.63, *SD* = 4.01).

### Agency Variables

#### 1:1 Staffing

**FIGURE 6:**
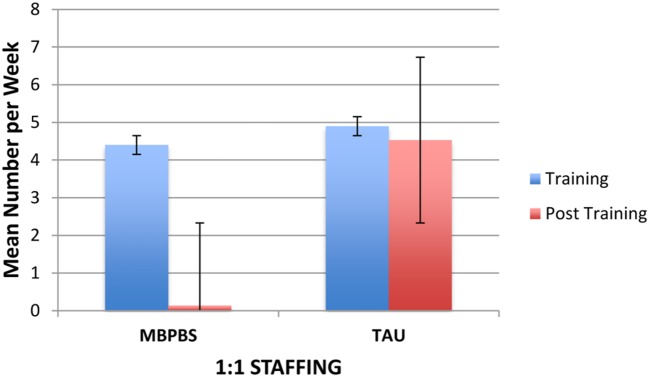
**Mean number of additional caregivers used for 1:1 staffing in the MBPBS and TAU conditions.** Error bars report standard error of the mean.

**Figure 6** shows the average 1:1 staffing (in addition to regular staffing) required for the care, safety and protection of staff and peers from individuals with IDD who were periodically aggressive. In the MBPBS condition, on average, 4.40 (*SD* = 1.78; range = 2–7) additional staff was required each week during Training and 0.13 (*SD* = 0.35; range = 0–1) during post-training. In the TAU condition, on average, 4.90 (*SD* = 1.45; range = 3–7) additional staff was required each week during Training and 4.53 (*SD* = 1.59; range = 3–9) during post-training. The difference between the two groups during the Training phase was not statistically significant, *t*(18) = 0.69, *p* = 0.50, but the difference between MBPBS vs. TAU post-training phases was statistically significant, *t*(58) = 14.80, *p* = 0.0001.

#### Staff Turnover

In terms of staff turnover, no caregiver in the MBPBS condition resigned due to injury and stress during the Training or post-training phases. Three caregivers resigned (all due to injury) during the Training phase in the TAU condition and 11 caregivers resigned (seven due to injury, four due to stress and injury) during the post-training phase. The difference between the MBPBS (*M* = 0.00, *SD* = 0.00) and TAU (*M* = 0.30, *SD* = 0.48) Training phases was not statistically significant, *t*(18) = 1.98, *p* = 0.06. During the post-training phase, the difference between the MBPBS (*M* = 0.00, *SD* = 0.00) and the TAU (*M* = 0.37, *SD* = 0.67) conditions was statistically significant, *t*(58) = 3.02, *p* = 0.004.

#### Cost Effectiveness

**Table 3** presents the cost effectiveness data for the MBPBS and TAU conditions. When compared to the TAU condition, the number of lost days of work due to staff injury was reduced by about 92% in the MBPBS condition, with a savings of $82,992.00. Commensurate with this savings, there was additional savings of $72,128.00 during the 40 weeks of the study in terms of additional costs of 1:1 staffing. The cost of medical and physical rehabilitation therapy services for the two injured staff during the MBPBS condition was $39,000.00 compared to $351,000.00 for the 18 staff injured during the TAU condition. While no additional costs were incurred in the MBPBS condition due to any staff turnover during the 40-week study period, the cost of training 14 new staff in the TAU condition was $17,680.00. The cost of providing alternate (temporary) staff for those who were in the MBPBS training was $41,440.00 compared to $42,560.00 in the TAU condition. Finally, the cost of providing the MBPBS training was $30,000.00 compared to $2,000.00 for TAU training. Overall, when compared to the TAU condition, there was a savings of $457,920.00 for an equivalent period during the MBPBS condition; that is, a savings of over 78%.

**Table 3:** Comparative costs for 40 weeks of MBPBS training condition (*n* = 37) compared to 40 weeks of TAU training condition (*n* = 38) for the human services variables.

	Cost Variables		Cost	
	MBPBS	TAU	MBPBS	TAU
Lost days of work and cost due to staff injury	42	536	$7,056.00	$90,048.00
Number of staff-days and cost of 1:1 staffing	96	740	$10,752.00	$82,880.00
Number and cost of staff needing medical and physical rehabilitation therapy	2	18	$39,000.00	$351,000.00
Number of staff resigned due to staff injury and training costs for new hires	0	14	$0.00	$17,680.00
Number of training days and cost of training	10	10	$30,000.00	$2,000.00
Cost of temporary staff during training	37	38	$41,440.00	$42,560.00
Total additional costs for the two time periods			$128,248.00	$586,168.00

**Total overall savings**				**$457,920.00**

## Discussion

Caregivers in mental health provide services for individuals with diverse abilities and challenging behaviors that often sap their mental and physical resources, and lead to stress and burnout. The traditional solution has been to teach caregivers specialized techniques, such as behavior analytic skills, that will help them to be more effective in managing the challenging behaviors of the individuals in their care (MacDonald, 2016; McIntyre and Neece, 2016). While this approach has been found effective, caregivers often work in situations where such skills alone may not be enough to curb their stress and burnout, because of the intensity and frequency of the challenging behaviors they face, multiple individuals engaging in challenging behaviors, the shortage of well-trained staff when the need arises, and the emotional toll of such work (Hastings, 2002; Allen et al., 2005; Crawford et al., 2010; Deveau and McGill, 2014).

An emerging approach involves not only enhancing the management skills of the caregivers in terms of the needs of the individuals they serve, but also teaching them self-management skills that enhance the caregivers’ psychological well-being, thereby making them more resilient in their work situation (Noone and Hastings, 2009, 2010; Brooker et al., 2013, 2014). For example, a series of exploratory proof-of-concept studies reported that adding mindfulness-based skills to caregivers previously or concurrently trained in PBS reduces their stress and burnout, as well as the challenging behaviors of those in their care (Singh et al., 2009, 2015, 2016b). Related studies have supported the notion that caregivers and their clients with disabilities mutually benefit when the caregivers are given training in mindfulness-based approaches (Brooker et al., 2013, 2014).

The present study strengthens the evidence-base for this approach. In a RCT, the current study demonstrated multiple beneficial effects of training caregivers in MBPBS compared to TAU in a congregate care long-term care facility for individuals with IDD. First, with regard to caregiver variables, the effects of the MBPBS training were evident in terms of statistically significant reductions in perceived psychological stress and job resignations due to stress and work-related injury. In addition, following training in MBPBS, staff greatly reduced using physical restraints and stat medicines contingent on the individuals’ aggressive behaviors. These findings confirm earlier reports of significant reductions in the use of physical restraints and emergency medications for individuals with aggressive behavior following training of caregivers in MBPBS (Singh et al., 2015, 2016b). Furthermore, Brooker et al. (2014) reported similar findings in terms of reduced staff use of PRN (“as needed”) medicines for behavior control, seclusions, and emergency chemical restraints following training in a mindfulness-based training program. Any reduction or elimination of the use of restrictive procedures indicates an enlightened approach to the care of people with diverse abilities, particularly those with IDD (Singh, 2016).

Second, with regard to the care recipient variable, reductions in aggressive incidents were significantly greater with the individuals receiving care from caregivers trained in MBPBS, than with those receiving care from caregivers trained in TAU. Caregivers in the MBPBS training condition did not receive instructions on how to manage the behavior of specific individuals in their care who engaged in aggressive behavior. In addition, existing behavior management plans for aggressive behavior were not reviewed or revised as a component of the MBPBS training condition. Given that the only difference between the MBPBS and TAU conditions was the nature of the training the caregivers received, the significantly reduced frequency of aggressive behavior evident in the MBPBS condition could be attributed to the personal change in the caregivers due to training in MBPBS. Similar reductions in incidents of verbal and physical aggression were reported in previous studies in which staff was trained in MBPBS (Singh et al., 2009, 2015, 2016b). All these findings suggest that training in MBPBS may change the very nature of the reciprocal interactions between the caregivers and the individuals in their care, moving them from a negative to a positive trajectory (Sameroff, 1995).

Third, with regard to agency variables, there was a significant reduction in the assignment of 1:1 staffing of individuals with aggressive behavior in the MBPBS condition when compared to the TAU condition. Indeed, when compared to the TAU condition, 1:1 staffing was rarely used following caregiver training in MBPBS. There was no staff turnover in the MBPBS condition compared to 14 in the TAU condition. Finally, the cost-effectiveness data showed substantial savings in the MBPBS condition when compared to the TAU condition. Costs were estimated for several standard variables (i.e., lost days of work due to staff injury, 1:1 staffing, treatment related costs for work-related staff injury, hiring and training of new staff, MBPBS and additional TAU training costs, temporary staff during training days) during the study period. There was an overall savings of 78.12% with the MBPBS training condition compared to the TAU training condition. Except for the costs of MBPBS training, which was much higher than for TAU, cost savings were realized on all other key variables in the MBPBS condition. Although the overall cost savings were somewhat less in the present study, they still aligned well with those from previous studies that reported savings 87.75% (Singh et al., 2015) and 89% (Singh et al., 2016b). Coupled with the enhanced psychological -being of the caregivers and reduced aggressive behavior of the care recipients, these cost savings suggest that MBPBS may be a clinically useful and financially viable training for caregivers.

The results of this RCT indicate that the traditional training provided in large facilities and community group homes for individuals with IDD may not be very effective in ameliorating the stress and burnout of the caregivers. While such training may enable caregivers to provide the required services detailed in each individual’s Individual Support Plan (ISP), the training is not very effective in assisting caregivers to effectively manage the behavior of individuals who engage in severe challenging behaviors, such as aggression, property destruction, and self-injury (Didden et al., 2016; Hoch et al., 2016). Furthermore, traditional training falls short in another respect—it does not teach caregivers how to successfully respond to workplace stress, compassion fatigue, and burnout. It is inevitable that caregivers will be stressed in large congregate care facilities for people with disabilities because of inherent demands in the job. Thus training should encompass strategies that enable caregivers to change their relationship to the job demands. Fortunately, mindfulness-based training does just that by teaching caregivers how to respond differently to the same daily work stresses, because they are typically not in position to change the nature of the job requirements (Hwang and Singh, 2016).

The MBPBS training enabled staff to respond in a calm and mindful way rather than to react negatively to the challenging behaviors of the individuals (Singh et al., 2016a). We suspect that disciplined meditation practice enables the caregivers to gradually change their relationship to their perceived mental and emotional experiences that arise when providing care to the individuals. This ability to step back and observe their thoughts and emotions as they occur results in cognitive, emotional and behavioral flexibility which helps them to respond more adaptively to difficult situations, thus reducing psychological stress and burnout (Shapiro et al., 2006). Indeed, it is likely that this metacognitive awareness enables caregivers to distance themselves from their reactive thoughts and emotions, and reperceive difficult situations as transient mental events (Safran and Segal, 1990). The Samatha and Kinhin meditations in the MBPBS training enable the caregivers to pay attention to the individuals with non-judgmental awareness, supporting them to work through their ISPs rather than by controlling their challenging behaviors. The meditations on the Four Immeasurables enable the caregivers to develop more equanimity in the face of daily work hassles and stresses, view the individuals and other staff with lovingkindness and compassion, and to demonstrate empathic joy as events unfold. Their training in adopting a beginner’s mind enables them to see more possibilities in terms of how to provide care to the individuals with challenging behaviors, buttressed by their training in seeing each individual and each event as if for the first time, without the baggage of history and emotional biases. For example, this mindset enables them to avoid reacting to the challenging behaviors of the individuals based on their premature cognitive commitment to control aggressive behavior through physical restraints and stat medications. While the actual mechanisms of the observed change due to training in mindfulness are yet to be explicated, it is increasingly evident that training staff in mindfulness produces behavioral changes in the staff as well as the individuals in their care (Eliassen et al., 2016).

In addition to its strengths as a RCT, this study has limitations as well. An important one is the issue of equivalence of the training in each of the two conditions of the study. It can be argued that the MBPBS condition included more training components than the TAU condition and, thus, should produce better outcomes. A standard control group design could not be used because congregate care facilities are required by policy to provide in-service training, thus restricting the comparison to the TAU condition. To address this possible limitation, future studies could evaluate the differential effects of MBPBS against standard behavioral training, the current gold standard of training in the care of individuals with challenging behaviors. Also, this study was executed in a congregate care facility for individuals who functioned at the severe to profound levels of IDD. Whether similar outcomes would be expected in other settings is open to speculation. Previous studies were executed in community group homes, and with individuals who had higher levels of intellectual functioning, factors that could likely produce similar or better results. In addition, it would be instructive to ask the caregivers in the MBPBS condition their views on what and how changes occurred as a consequence of their participation in the meditation practices. Furthermore, future research should investigate the importance of the mindfulness trainer’s personal meditation practice and the authenticity of the teachings as factors that may impact outcomes (Carmody and Baer, 2009; McCown et al., 2010).

Mindfulness-Based Positive Behavior Supports provides a paradigm shift in terms of how caregivers and care recipients can be mutually engaged in enhancing the quality of their lives. This model is based on multiple theoretical underpinnings, including Sameroff’s (1995) bidirectional transactions, mindfulness (Shonin et al., 2015), PBS (Morris and Horner, 2016), and patient engagement (Institute of Medicine, 2011; Barello et al., 2014; Graffigna et al., 2016), which is known as person-centered planning in the field of IDD (Ratti et al., 2016). In their lexicographic literature review, Barello et al. (2014) noted that the concept of patient engagement has changed over time, and still remains an elusive concept in clinical practice and health care policy. Coulter (2011, p. 10) has suggested that when patients and caregivers work together on patient engagement, they “promote and support active patient and public involvement in health and healthcare and… strengthen their influence on healthcare decisions, at both the individual and collective levels.” The MBPBS model is grounded in mutual engagement of both the caregiver and care recipient, and by changing the behavior of one of the pair, the behavior of the other changes because of the bidirectional transactions that occur (Sameroff, 1995; Singh and Singh, 2001). Training caregivers in MBPBS not only elicits and enhances the individual’s activation—his or her ability and willingness to self-manage challenging behaviors—but also promotes engagement in positive behavior, as evidenced in the present and previous studies (Singh et al., 2015, 2016b).

There are several implications of this study. First, these findings need further validation in terms of the effects of MBPBS against other proven techniques, such as behavior analytic strategies, as well as replications by other research teams. Second, the effects of MBPBS need to be assessed in different care settings, such as institutions, group homes, family homes, and within the larger community settings. Third, the effects of MBPBS need to be further evaluated with parents and teachers of individuals with IDD, caregivers of other populations, such as the elderly and individuals with neurodevelopmental disorders. These kinds of studies will help refine the MBPBS model and delineate the boundary conditions for its effectiveness. Furthermore, future research should also focus on dissemination, especially given the finding that few evidence-based practices are actually implemented in real-world settings (Katz, 2010). The challenge is to make MBPBS not too intensive or effortful, but still produces replicable and long-lasting effects at a reasonable cost (Glasgow et al., 2003; Singh, 2016).

In sum, this RCT provides further evidence that MBPBS may be a viable approach to caregiver training to improve the psychological well-being of both caregivers and the care recipients. This study showed that, when provided training in MBPBS, caregivers could significantly reduce their own stress levels and the use of restrictive procedures (e.g., physical restraints and emergency medications) when confronted with challenging behaviors by individuals with intellectual disabilities. When caregivers changed the nature of their care, the individuals reduced their aggressive behaviors and obviated the need for 1:1 staffing. Finally, the cost effectiveness data suggest that agencies may want to implement MBPBS training not only because of the benefits for the caregivers and care recipients, but also for financial savings.

## Author Contributions

NS: designed and executed the study, assisted with the data analyses, and wrote the paper. GL: collaborated with the design and writing of the study. BK: analyzed the data and wrote part of the results. JC: collaborated with the design and writing of the study. AW: collaborated in the writing and editing of the final manuscript.

## Conflict of Interest Statement

NS is the developer of the MBPBS program. All the other authors declare that the research was conducted in the absence of any commercial or financial relationships that could be construed as a potential conflict of interest.
